# Long-term benefit of DAAs on gut dysbiosis and microbial translocation in HCV-infected patients with and without HIV coinfection

**DOI:** 10.1038/s41598-023-41664-7

**Published:** 2023-09-02

**Authors:** Natthaya Chuaypen, Thananya Jinato, Anchalee Avihingsanon, Intawat Nookaew, Yasuhito Tanaka, Pisit Tangkijvanich

**Affiliations:** 1 Department of Biochemistry, Center of Excellence in Hepatitis and Liver Cancer, Faculty of Medicine, Chulalongkorn University, Bangkok, Thailand; 2 Doctor of Philosophy Program in Medical Sciences, Graduate Affairs, Faculty of Medicine, Chulalongkorn University, Bangkok, Thailand; 3 The HIV Netherlands Australia Thailand Research Collaboration (HIV-NAT), Bangkok, Thailand; 4 Department of Biomedical Informatics, College of Medicine, University of Arkansas for Medical Sciences, Little Rock, AR USA; 5 Division of Integrated Medical and Pharmaceutical Sciences, Department of Gastroenterology and Hepatology, Faculty of Life Sciences, Kumamoto University, Kumamoto, Japan

**Keywords:** Molecular biology, Molecular medicine

## Abstract

Long-term effect of Direct-acting antivirals (DAAs) on gut microbiota, short-chain fatty acids (SCFAs) and microbial translocation in patients with hepatitis C virus (HCV) infection who achieve sustained virological response (SVR) were limited. A longitudinal study of 50 patients with HCV monoinfection and 19 patients with HCV/HIV coinfection received DAAs were conducted. Fecal specimens collected at baseline and at week 72 after treatment completion (FUw72) were analyzed for 16S rRNA sequencing and the butyryl-CoA:acetateCoA transferase (BCoAT) gene expression using real-time PCR. Plasma lipopolysaccharide binding protein (LBP) and intestinal fatty acid binding protein (I-FABP) were quantified by ELISA assays. SVR rates in mono- and coinfected patients were comparable (94% vs. 100%). The improvement of gut dysbiosis and microbial translocation was found in responders but was not in non-responders. Among responders, significant restoration of alpha-diversity, BCoAT and LBP were observed in HCV patients with low-grade fibrosis (F0–F1), while HCV/HIV patients exhibited partial improvement at FUw72. I-FABP did not decline significantly in responders. Treatment induced microbiota changes with increasing abundance of SCFAs-producing bacteria, including *Blautia, Fusicatenibacter, Subdoligranulum and Bifidobacterium*. In conclusion, long-term effect of DAAs impacted the restoration of gut dysbiosis and microbial translocation. However, early initiation of DAAs required for an alteration of gut microbiota, enhanced SCFAs-producing bacteria, and could reduce HCV-related complications.

## Introduction

Chronic hepatitis C virus (HCV) infection is a global public health, affecting 60 million people, and could lead to the development of cirrhosis and hepatocellular carcinoma (HCC)^1^. Moreover, an estimated 2.3 million people with human immunodeficiency virus (HIV) coinfected with HCV has an increased risk of liver-related complications, including higher rates of cirrhosis, end-stage liver disease and HCC^2^. Currently, direct-acting antivirals (DAAs) are the standard of care for patients with HCV monoinfection and HCV/HIV coinfection with sustained virological response (SVR) rates greater than 95%^3^. It has been shown that HCV eradication by DAAs is associated with regression of liver fibrosis and a significantly reduced risk of cirrhosis and HCC. Additionally, successful HCV clearance results in decreasing various extra-hepatic manifestations including diabetes mellitus, stroke and chronic kidney disease^4^. Recent data have also demonstrated a survival benefit of HCV therapy, even in patients with mild liver disease^3^. Thus, it is recommended that early therapy with DAAs should be applied for any HCV-infected individuals to prevent complications beyond liver disease.

Gut dysbiosis, characterized by the imbalance of diversity and composition of gut microbiota, plays an important role in the natural course of various chronic liver diseases^5, 6^. Aberrant gut microbiota, together with alteration in microbial metabolites such as short-chain fatty acids (SCFAs) and intestinal barrier dysfunction could eventually accelerate liver injury through the gut-liver axis^7^. In chronic HCV infection, it was shown that gut dysbiosis existed even in mild hepatitis and appeared to exhibit more changes in progressive liver disease^8, 9^. Moreover, bacterial translocation resulting from intestinal barrier dysfunction was also detected in the absence of significant fibrosis or cirrhosis, indicating that gut barrier dysfunction might occur during early stages of chronic HCV infection^10, 11^.

Emerging evidence have indicated that antiviral therapy could potentially improve gut dysbiosis in patients achieving SVR^12, 13^. In line with these results, our recent study demonstrated the short-term effect of DAAs (12-weeks after therapy) in restoration of microbial dysbiosis after HCV eradication in patients with HCV mono- and HCV/HIV coinfection, particularly among those who had early fibrosis stages at baseline^14^. It was also demonstrated that surrogate microbial translocation markers, such as lipopolysaccharide binding protein (LBP), were significantly reduced after anti-HCV treatment^15^. Despite these observations, the relationships between gut dysbiosis and bacterial translocation in patients with chronic HCV infection before and after DAA therapy are not well-characterized. Moreover, most available data regarding gut microbiota and related biomarkers are limited to relatively short duration of follow-up. Thus, more data on long-term studies after achieved SVR are required to determine whether microbiome changes are maintained over time with regard to the extent in liver fibrosis and HIV status.

In this prospective longitudinal study, we aimed at investigating the impact of DAAs on microbiota restoration in Thai patients with HCV mono- and HCV/HIV coinfection at 72-weeks after achieving HCV eradication, Moreover, we evaluated the alteration of microbial communities among these patients in association with the changes of SCFAs and microbial translocation markers after attaining SVR.

## Results

### Baseline characteristics of patients

Sixty-nine HCV-infected patients who followed-up for 72 weeks after DAAs treatment completion (FUw72) were included in this present study. Baseline characteristics of 50 patients with HCV monoinfection, 19 patients with HCV/HIV coinfection and 20 healthy controls were summarized in Table 1. The HCV and HCV/HIV groups had significantly higher levels of aspartate aminotransferase (AST) and alanine aminotransferase (ALT) than healthy controls. Compared to the HCV/HIV group, patients with HCV monoinfection had lower levels of serum creatinine but had greater BMI and higher proportion of F2–F4. There were no differences between groups of patients regarding other baseline parameters. Sustained virological response rates (SVR) was also comparable between the HCV and HCV/HIV groups (94% vs. 100%).

**Table 1:** Baseline characteristics of patients.

Characteristics	Healthy controls (n = 20)	HCV monoinfection (n = 50)	HCV/HIV coinfection (n = 19)	*P*-value
Male	9 (45.0)	36 (72.0)	14 (73.7)	0.072
Age (years)	48.8 ± 8.7	50.0 ± 10.7	43.6 ± 7.2	0.084
BMI (kg/m^2^)	22.9 ± 2.6	24.7 ± 3.5	22.4 ± 4.5	0.007*
Hemoglobin (g/dL)		14.3 ± 1.2	14.8 ± 1.6	0.194
Platelets (10^9^/L)		200.1 ± 71.0	213.7 ± 77.6	0.744
WBC count (10^3^/μL)		5.7 ± 1.3	6.0 ± 1.2	0.391
AST (IU/L)	21.6 ± 5.8	53.3 ± 38.5	43.4 ± 18.7	< 0.001**
ALT (IU/L)	20.6 ± 6.0	65.1 ± 54.8	51.5 ± 24.2	< 0.001*
Creatinine (mg/dL)		0.8 ± 0.2	1.0 ± 0.2	0.036*
eGFR (mL/min/1.73 m^2^)		99.3 ± 16.5	96.7 ± 15.6	0.281
Log_10_ HCV RNA (IU/mL)		6.3 ± 0.6	6.5 ± 0.5	0.594
Magnetic resonance elastography (kPa)		3.0 ± 1.0	2.6 ± 0.9	0.057
Liver fibrosis stage by MRE				
F0–F1		23 (48.9)	15 (78.9)	0.026*
F2–F4		24 (51.1)	4 (21.1)	
SVR rate (%)		47 (94.0)	19 (100)	0.275
Mode of HIV transmission	–	–		
Men who have sex with men			2 (10.5)	–
Intravenous drug users			13 (68.4)	
Heterosexual			4 (21.1)	
Antiretroviral therapy	–	–		
NNRTI-based			13 (68.4)	
Protease inhibitor-based			4 (21.1)	
Others			2(10.5)	
HIV viral suppression (< 50 copies/mL)	–	–	19 (100)	–
CD4 + T-cell count (cells/mm^3^)	–	–	442.7 ± 148.3	–
CD8 + T-cell count (cells/mm^3^)	–	–	643.4 ± 280.7	–

Data as shown in n (%), mean ± SD; n (%), median ± IQR

*HCV* Hepatitis C virus; *HIV* Human immunodeficiency virus; *WBC* White blood cell; *BMI* Body mass index; *AST* Aspartate aminotransferase; *ALT* Alanine aminotransferase; *eGFR* Estimated glomerular filtration rate; *MRE* Magnetic resonance elastography; *SVR* Sustained virological response; *NNRTI* Nonnucleoside reverse transcriptase inhibitor

*P < 0.05, **P < 0.001

### Microbial diversity before and long-term follow-up after DAA therapy

**Figure 1:**
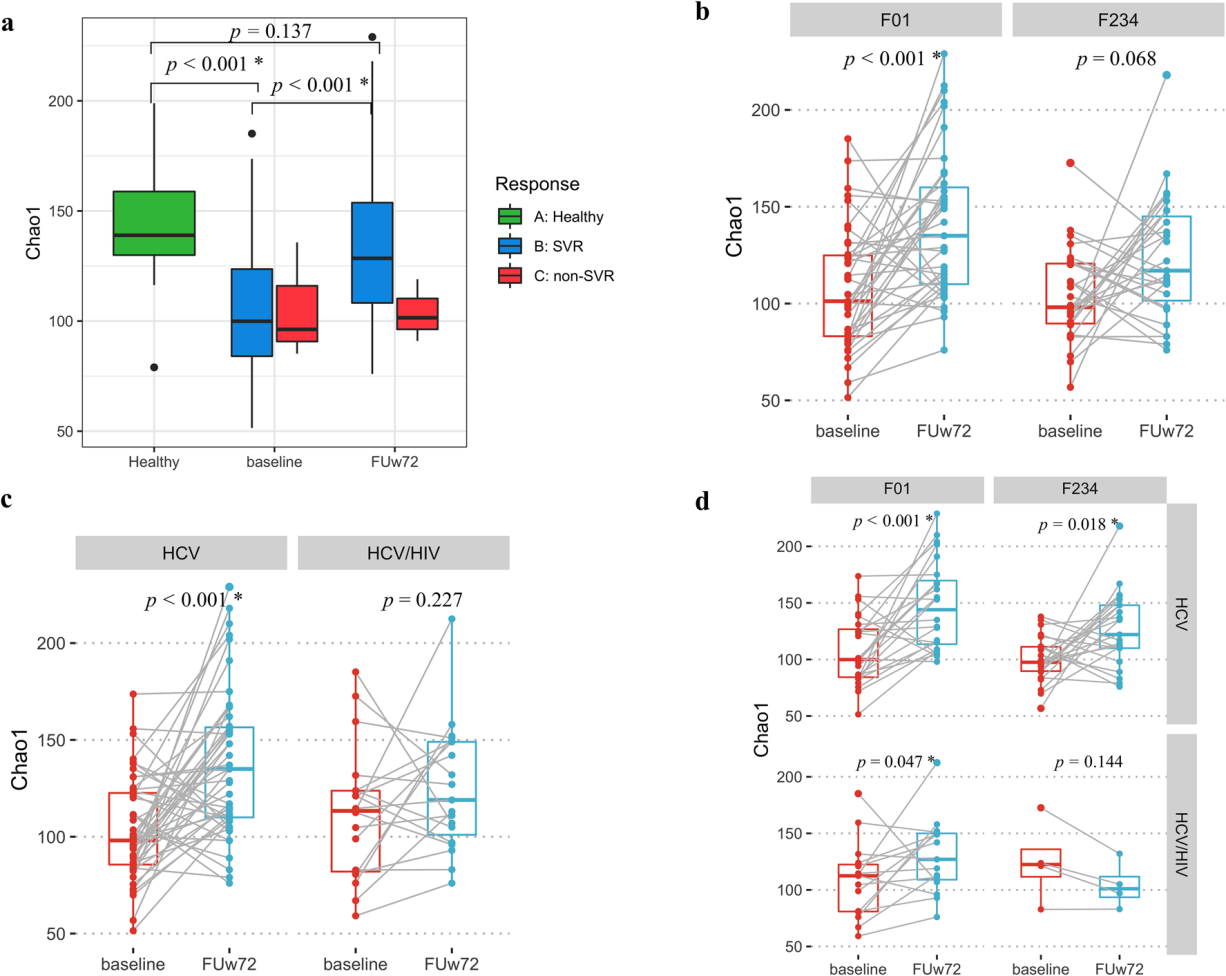
Richness of gut microbiome (Chao1) at baseline and follow-up week-72 (FUw72) in (**a**) healthy controls, patients with SVR and non-SVR, (**b**) F0–F1 versus F2–F4 fibrosis stages, (**c**) HCV monoinfection versus HCV/HIV coinfection, (**d**) subgroup analysis of fibrosis stages and HIV status.

Our previous study demonstrated that DAAs treatment improved microbial diversity at SVR12^14^. We next sought to determine the community structure of gut microbiota at baseline compared to FUw72. At baseline, richness (Chao1) of the patient’s groups were significantly lower than that of the healthy control group (median ± IQR, 99.38 ± 39.50 vs. 140.49 ± 32.32, Fig. 1a, Mann–Whitney U test, *P* < 0.001), whereas alpha diversity (Simpson) did not show statistical difference between patient and healthy control groups (Supplementary Fig. S1). Compared with baseline, significant improvement of Chao1 and Simpson were observed at FUw72 in patients achieved SVR (Fig. 1a, Chao1; 128.5 ± 46.50 vs. 99.91 ± 40.05, Wilcoxon Signed Ranks Test, *P* < 0.001 and Supplementary Fig. S1 Simpson; 0.97 ± 0.01 vs. 0.95 ± 0.02, *P* < 0.001), but were not found in patients with non-SVR. These data suggested that increased bacteria richness and diversity remained stable overtime in patients who achieved SVR.

Regarding fibrosis stages, we observed significant difference in Chao1 between baseline and FUw72 only in patients with mild fibrosis (F0–F1) (Fig. 1b, 101.21 ± 42.33 vs. 135.00 ± 53.00, Wilcoxon Signed Ranks Test, *P* < 0.001), but did not observe the difference in the significant fibrosis to cirrhosis (F2–F4) group (98.10 ± 32.19 vs. 117.00 ± 50.00, *P* = 0.068). Compared with baseline, overall Chao1 index at FUw72 showed significantly increased only in patients with HCV monoinfection (Fig. 1c, 98.10 ± 38.54 vs. 135.00 ± 47.00, *P* < 0.001), while there was no significant difference in patients with HCV/HIV coinfection (Fig. 1c, 113.35 ± 42.64 vs. 119.00 ± 52.00, *P* = 0.227). Interestingly, in subgroup analysis, improvement of Chao1 at FUw72 was found in both the HCV monoinfection and HCV/HIV coinfection groups who had F0–F1 fibrosis stage (Fig. 1d, *P* < 0.001 and *P* = 0.047, respectively), while the improvement of Chao1 in patients with F2–F4 was found only in patient with HCV monoinfection (*P* = 0.018). These results suggested that patients with HCV monoinfection had significant improvement of richness of bacteria, regardless of baseline fibrosis stages. Additionally, richness and evenness in terms of Simpson was significantly increased at SVR72 compared with baseline in both F0–F1 and F2–F4 groups, HCV monoinfection and HCV/HIV coinfection groups and in subgroup analysis of viral and fibrosis status (Supplementary Fig. S1b–d).

**Figure 2:**
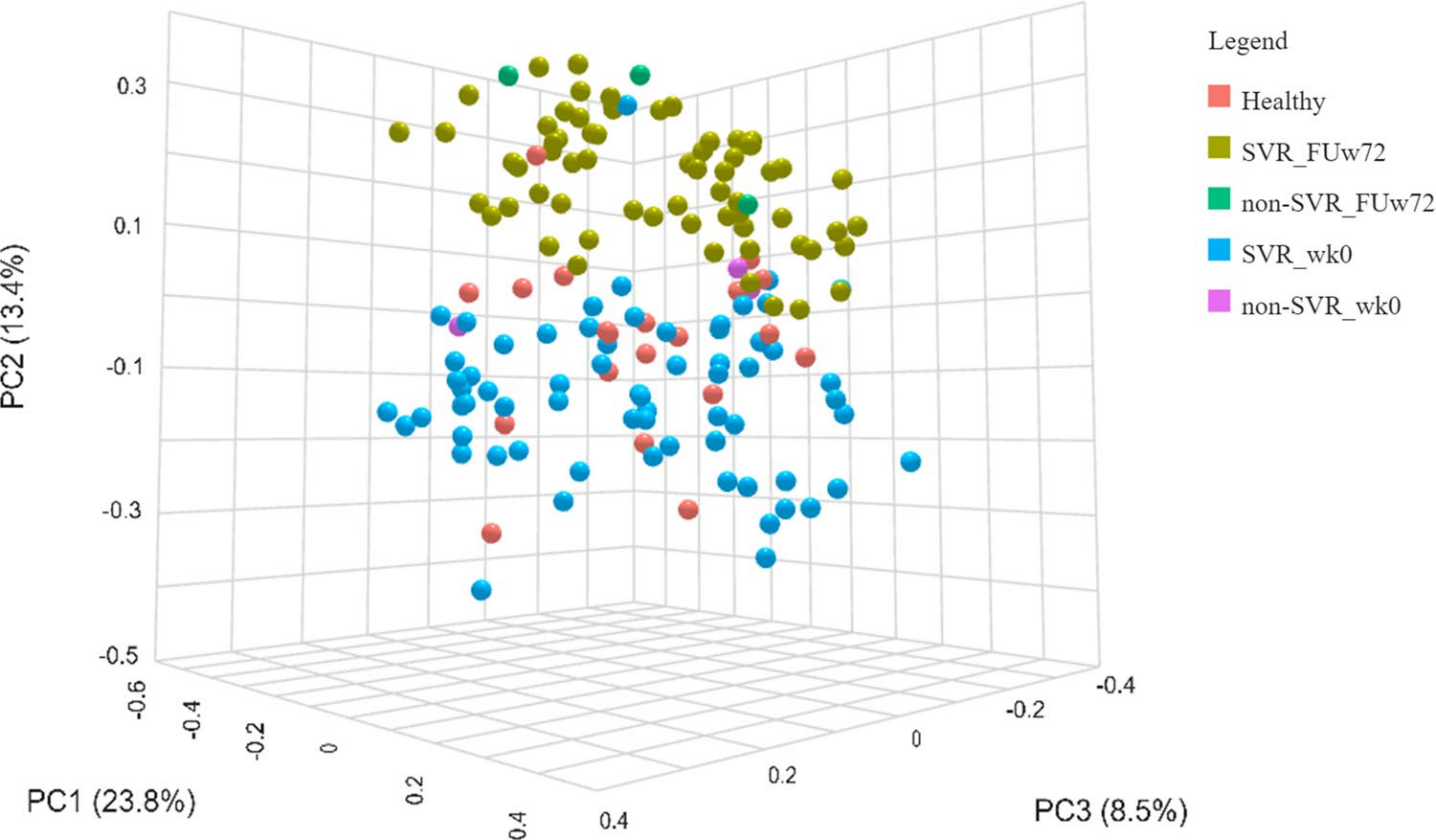
Beta-diversity (Bray–Curtis distance) of healthy controls, HCV monoinfection and HCV/HIV coinfection, SVR, and non-SVR at baseline and follow-up week-72 (FUw72).

Next, we determined whether treatment changed the microbiota composition in terms of beta-diversity. Principal Coordinates Analysis (PCoA) plot based on Bray–Curtis dissimilarity indices was performed. Our result demonstrated that microbial community composition of patients at FUw72 was significantly differences from baseline and healthy controls (Fig. 2, PERMANOVA, *P* = 0.001). These results indicated that bacterial community at follow-up still clustered distinctly from those of baseline and healthy controls.

### Microbiota composition and differential abundance before and long-term follow-up after DAA therapy

**Figure 3:**
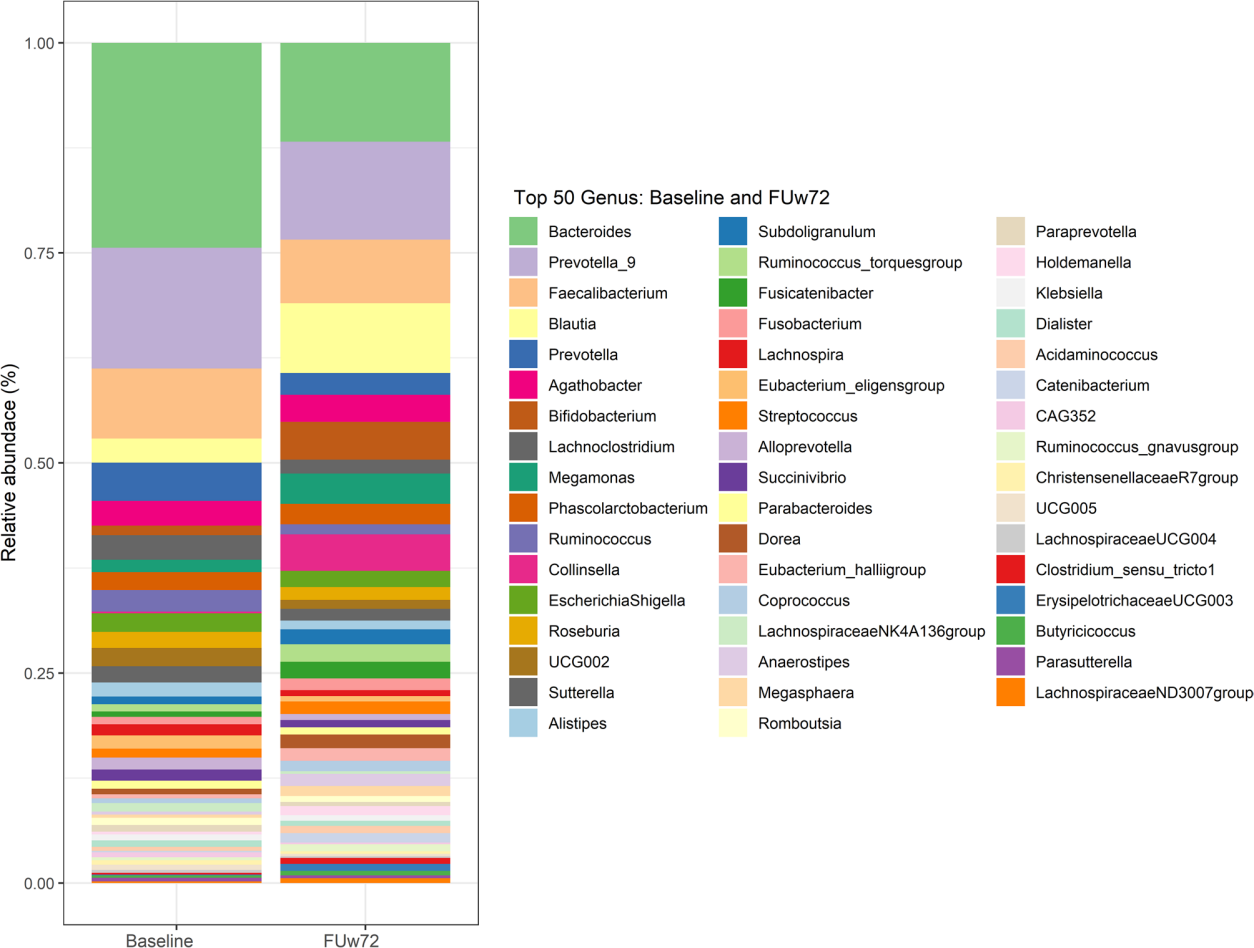
Relative abundance of top 50 abundant genera at baseline and follow-up week-72 (FUw72).

To investigate long-term alteration of gut microbiota composition following DAAs, we compared relative abundance of top 50 genera in patients at baseline and FUw72 (Fig. 3). Further details were available in Supplementary Table S2.

As shown in Table 2, we identified the difference genera of bacteria (based on relative abundance at median ≥ 1%) between patients with SVR and non-SVR. Among the SVR group, there was a significant increase in 14 SCFAs-producing bacteria such as *Blautia*, *Bifidobacterium*, *Subdoligranulum* and *Dorea*, most of which were SCFAs-producing bacteria. In contrast, 5 genera were decreased at FUw72, including *Bacteroides, Lachnoclostridium, Sutterella, Lachnospira and Eubacterium eligens group*. In the non-SVR group, significant difference in bacterial genera between baseline and FUw72 was not observed.

**Table 2:** Relative abundance (≥ 1%) at genus levels in patients with SVR and non-SVR at baseline and FUw72.

Genus	SVR (n = 66)		*P*-values	Non-SVR (n = 3)		*P*-values
	Baseline	FUw72		Baseline	FUw72	
Increasing bacteria in SVR group						
*Blautia*	0.018 ± 0.02	0.073 ± 0.04	< 0.001**	0.017	0.069	0.109
*Bifidobacterium*	0.002 ± 0.01	0.028 ± 0.06	< 0.001**	0.006	0.104	0.109
*Subdoligranulum*	0.003 ± 0.01	0.012 ± 0.02	< 0.001**	0.025	0.014	0.593
*Dorea*	0.005 ± 0.00	0.014 ± 0.01	< 0.001**	0.006	0.013	0.593
*Megamonas*	0.001 ± 0.01	0.007 ± 0.06	0.002*	0.019	0.059	1.000
*Collinsella*	0.000 ± 0.00	0.043 ± 0.02	< 0.001**	0.000	0.019	0.285
*Ruminococcus torques group*	0.004 ± 0.01	0.014 ± 0.02	< 0.001**	0.011	0.022	1.000
*Fusicatenibacter*	0.002 ± 0.01	0.013 ± 0.02	< 0.001**	0.014	0.031	0.109
*Streptococcus*	0.001 ± 0.00	0.006 ± 0.01	< 0.001**	0.001	0.013	0.285
*Eubacterium hallii group*	0.003 ± 0.00	0.014 ± 0.01	< 0.001**	0.003	0.019	0.109
*Coprococcus*	0.003 ± 0.01	0.010 ± 0.02	< 0.001**	0.009	0.012	1.000
*Anaerostipes*	0.001 ± 0.00	0.006 ± 0.01	< 0.001**	0.002	0.014	0.109
*Megasphaera*	0.000 ± 0.00	0.000 ± 0.01	0.006*	0.004	0.024	1.000
*Holdemanella*	0.000 ± 0.00	0.000 ± 0.02	0.004*	0.000	0.032	0.180
*Decreasing bacteria in SVR group*						
*Bacteroides*	0.170 ± 0.33	0.090 ± 0.14	< 0.001**	0.087	0.155	0.593
*Lachnoclostridium*	0.013 ± 0.02	0.009 ± 0.02	0.034*	0.024	0.012	0.109
*Sutterella*	0.015 ± 0.02	0.010 ± 0.01	0.036*	0.023	0.014	0.593
*Lachnospira*	0.007 ± 0.01	0.004 ± 0.01	0.033*	0.016	0.008	0.285
*Eubacterium eligens group*	0.006 ± 0.01	0.002 ± 0.01	0.002*	0.011	0.000	0.180

Data shown in median ± IQR

SVR, Sustained virological response rates; FUw72, *follow*-up week-72

*P < 0.05, **P < 0.001 (Wilcoxon Signed Ranks Test)

In the HCV monoinfection group, significant differences in the abundance of enriched 8 genera were observed, while 2 genera were depleted. In the HCV/HIV coinfection group, 9 genera were significantly increased, and 3 genera were reduced. Additionally, SCFAs-producing bacteria such as *Subdoligranulum*, *Fusicatenibacter,* and *Collinsella* were significantly higher at FUw72 in patients with HCV monoinfection than those with HCV/HIV coinfection (Supplementary Tables S3, S4). These results might suggest that successful DAA treatment exhibits long-term impacts on gut microbiota, especially an increase in SCFAs-producing bacteria.

### Butyryl-CoA:acetateCoA transferase (BCoAT) gene level before and long-term follow-up after DAA therapy

**Figure 4:**
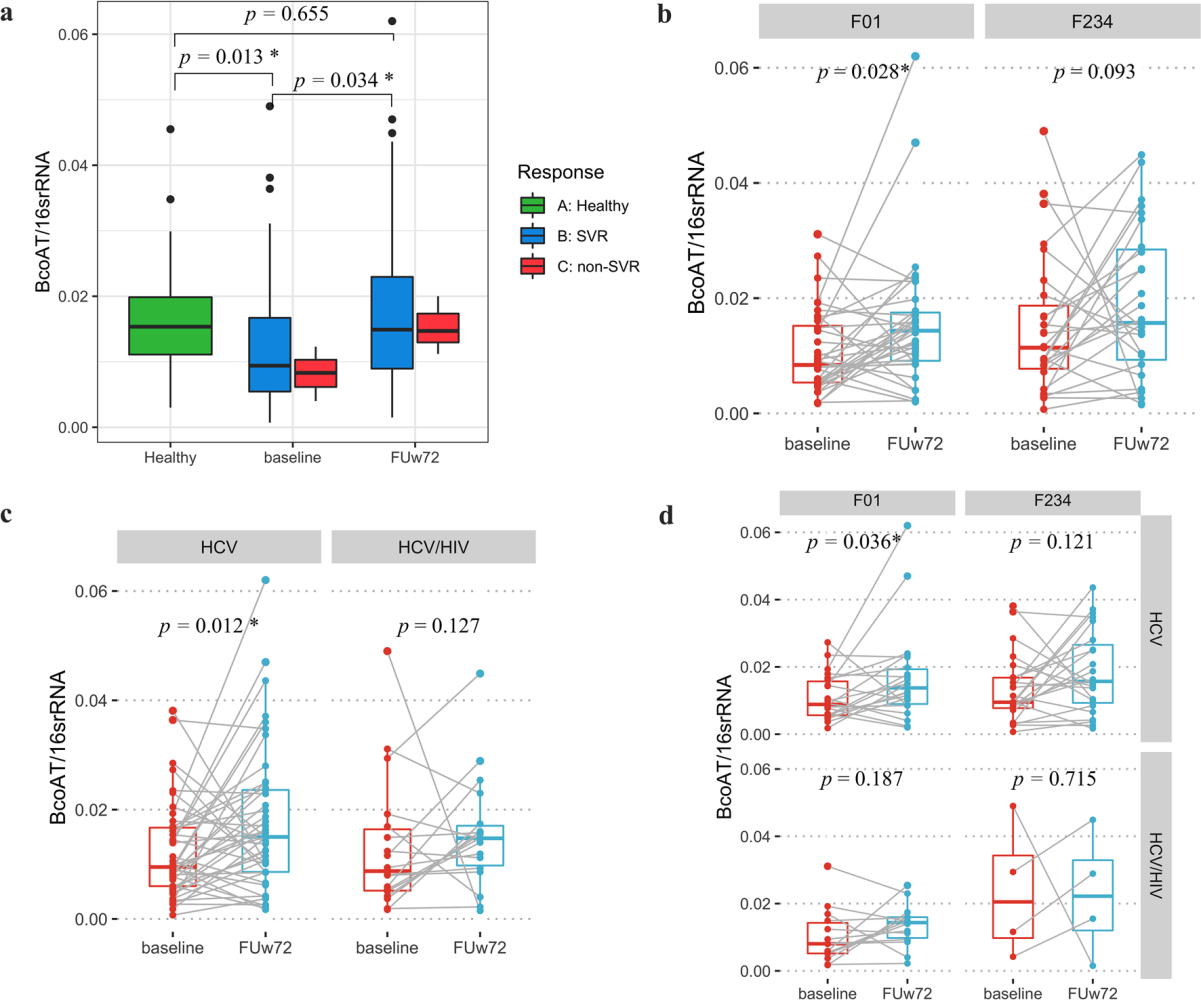
The levels of BCoAT gene in patients at baseline and follow-up week-72 (FUw72) in (**a**) healthy controls, patients with SVR and non-SVR, (**b**) F0–F1 versus F2–F4 fibrosis stages, (**c**) HCV monoinfection versus HCV/HIV coinfection, (**d**) subgroup analysis of fibrosis stages and HIV status.

Based on the differential abundance of SCFAs-producing bacteria, we next investigated the expression of butyrate gene in fecal samples of patients and healthy controls. In this regard, the BCoAT gene was determined by real-time PCR. Our result demonstrated that a significant increase in BCoAT expression was observed at FUw72 compared with baseline (Median ± IQR, 0.015 ± 0.014 vs. 0.009 ± 0.011, Fig. 4a, Wilcoxon Signed Ranks Test, *P* = 0.034) and its level was comparable with that of healthy individuals (0.015 ± 0.014 vs. 0.015 ± 0.012, Mann–Whitney U test, *P* = 0.655), However, such result was not detected in patients with non-SVR (*P* = 0.285).

Based on fibrosis stages in patients with SVR, patients with low degree of fibrosis (F0–F1) showed significantly increase in BCoAT expression in microbiota at FUw72 compared with baseline (Fig. 4b, 0.014 ± 0.008 vs. 0.008 ± 0.005, Wilcoxon Signed Ranks Test, *P* = 0.028), while there was no difference in patients with significant fibrosis (F2–F4) (Fig. 4b, *P* = 0.093). Moreover, patients with HCV monoinfection had significantly higher level of BCoAT at FUw72 than baseline (Fig. 4c, 0.015 ± 0.015 vs. 0.010 ± 0.011, *P* = 0.012). In contrast, there was no significant difference in BCoAT expression in patients with HCV/HIV coinfection group (*P* = 0.127). In subgroup analysis, the significant increase in expression of BCoAT was only observed in HCV monoinfected patients who had low degree of fibrosis (F0–F1) (Fig. 4d, *P* = 0.036), while there was no significant difference in HCV monoinfected patients with F2–F4, as well as in all patients with HCV/HIV coinfection. These results suggested that enrichment of SCFAs-producing bacteria could increase butyrate gene expression in fecal samples, particularly among HCV monoinfected patients with low fibrosis stage.

### Reduction of microbial translocation and enterocytes damage biomarkers after long-term follow-up after DAA therapy

**Figure 5:**
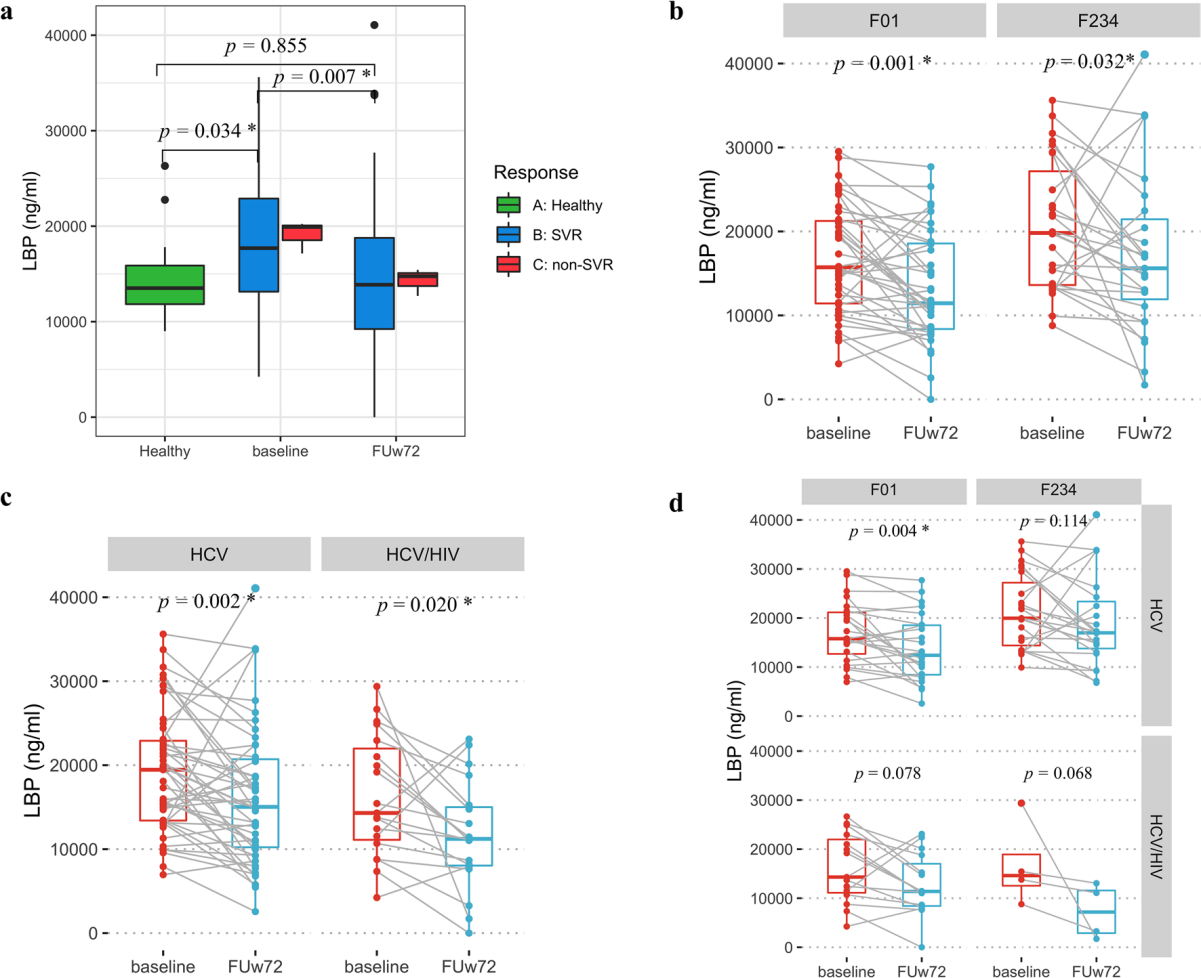
The levels of plasma LBP in patients at baseline and follow-up week-72 (FUw72) in (**a**) healthy controls, patients with SVR and non-SVR, (**b**) F0–F1 versus F2–F4 fibrosis stages, (**c**) HCV monoinfection versus HCV/HIV coinfection, (**d**) subgroup analysis of fibrosis stages and HIV status.

Next, we investigated whether DAAs could improve surrogate markers of microbial translocation (LBP) and intestinal damage (I-FABP). The plasma levels of LBP and I-FABP were determined at baseline and FUw72 in HCV-infected patients. Overall, LBP level at FUw72 showed significantly lower than baseline in patients achieving SVR (Median ± IQR, 13,867.79 ± 10,058.99 vs. 17,701.93 ± 9,889.89, Fig. 4a, Wilcoxon Signed Ranks Test, *P* < 0.007), which was comparable with heathy controls (13,867.79 ± 10,058.99 vs. 13,514.79 ± 5,073.96, Mann–Whitney U test, *P* = 0.855). While there was no significant difference in patients with non-SVR (Fig. 5a, *P* = 0.109).

Based on fibrosis status, LBP level was significantly lower in both patients with F0–F1 and F2–F4 at FUw72 compared with baseline (Fig. 5b, 11,449.48 ± 10,482.63 vs. 15,730.77 ± 10,120.19 ng/mL, *P* = 0.001 and 15,610.58 ± 11,376.31 vs. 19,817.31 ± 15,929.19 ng/mL, *P* = 0.032, respectively). Regarding HIV status, there was a significant decrease in LBP level at FUw72 in patients with HCV monoinfection and HCV/HIV coinfection (Fig. 5c, 15,033.65 ± 10,993.51 vs. 19,456.73 ± 9,720.69 ng/mL, *P* = 0.002 and 11,223.00 ± 7317.08 vs. 14,306.62 ± 12,264.81 ng/mL, *P* = 0.020, respectively). In subgroup analysis, however, only HCV-monoinfected patients with F0–F1 had significantly lower LBP level at FUw72 in comparison with baseline (Fig. 5d, 12,398.96 ± 10,468.59 vs. 15,776.54 ± 9,573.32 ng/mL, *P* = 0.004).

To investigate whether the effect of DAA therapy on reducing markers of microbial translocation (LBP) was associated with improvement of gut diversity, we further analyzed the correlation between LBP and BCoAT gene expression. Our result showed that the alteration in LBP was negatively correlated with the change in BCoAT gene expression (Spearman, r = -0.315, *P* = 0.011), especially in patients with HCV monoinfection (r = − 0.393, *P* = 0.006). However, there was no such correlation in patients with HCV/HIV coinfection (r = − 0.038, *P* = 0.880) as shown in Supplementary Fig. S2a–c.

**Figure 6:**
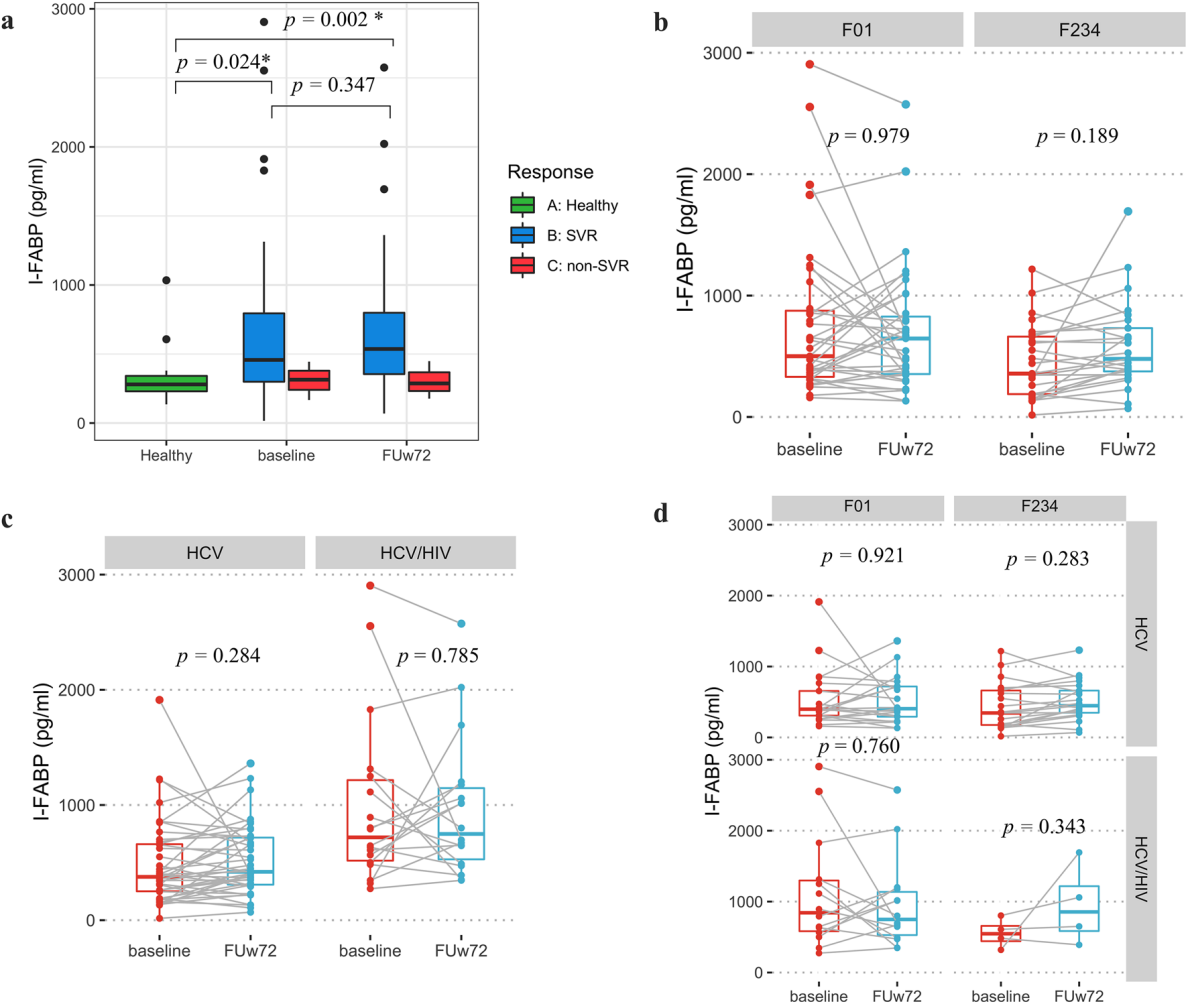
The levels of plasma I-FABP in patients at baseline and follow-up week-72 (FUw72) in (**a**) healthy controls, patients with SVR and non-SVR, (**b**) F0–F1 versus F2–F4 fibrosis stages, (**c**) HCV monoinfection versus HCV/HIV coinfection, (**d**) subgroup analysis of fibrosis stages and HIV status.

Regarding intestinal damage marker at baseline and FUw72, overall pateints with SVR had a significantly higher level of I-FABP than those of healthy controls (Fig. 6a, 457.10 ± 518.57 vs. 279.71 ± 138.57, Mann–Whitney U test, *P* = 0.024 and 536.34 ± 448.00 vs. 279.71 ± 138.57, *P* = 0.002, respectively). Moreover, no significant difference in I-FABP level was found between before and after DAAs in all subgroup analysis in terms of fibrosis status and HIV infection (Fig. 6b, c, d). These results could suggest that successful DAAs therapy did not have an effect on the change in intestinal damage marker.

## Discussion

Antiviral therapy could alleviate gut dysbiosis in patients with HCV monoinfection and HCV/HIV coinfection, who achieved SVR in short follow-up period. However, it is unclear whether HCV eradication leads to a sustained restoration of gut microbial communities, changes of gut SCFAs production and microbial translocation, in extended period of time. In this report, we demonstrated in a large prospective cohort of patients that most individuals with HCV infection experienced an improvement of microbial diversity in long-term, post-SVR following DAA therapy. However, these favorable changes after viral eradication were mainly observed in responders with baseline low-grade fibrosis (F0–F1) compared with those with more advanced fibrotic stages (F2–F4). Our data were in agreement with previous reports demonstrating that HCV eradication improved gut dysbiosis, particularly among those without advanced liver disease^13, 16, 17^. Additionally, a significant restoration of gut microbial communities was seen mainly in HCV mono- as compared to HCV/HIV coinfected individuals. These data highlight the significance of early treatment in the natural history of chronic HCV infection and underline the complexity of HIV coinfection affecting gut dysbiosis improvement in patients with HCV infection.

In this study, we demonstrated that successful DAA therapy was able to restore gut microbiota diversity and composition. In contrast, the alleviation of microbial dysbiosis was not observed in non-responders, considering there were a small number of patients. Additionally, shifts in microbial community structure resulting from HCV clearance would elicit a significant change in its functions. Specifically, effective DAAs could increase abundance of beneficial bacteria, particularly SCFA-producing bacteria. Similarly, bacterial gene coding BCoAT, a reliable semiquantitative assay for fecal butyrate was also shown to be significantly increased in individuals achieved SVR. SCFAs, particularly acetate, propionate, and butyrate, are the main gut metabolites that are critical for maintaining intestinal barrier integrity, preventing microbial translocation and further reduced chronic inflammation^18^. In addition, SCFAs interact with epithelial cells or immune cells leading to essential anti-inflammatory and immunomodulatory effects. Indeed, gut dysbiosis with decreased SCFA-producing bacteria and depleted SCFAs are frequently reported in various metabolic disorders including type 2 diabetes and obesity^19^. Moreover, it has been shown that the reduction of SCFAs-producing bacteria is associated with liver inflammation and steatosis in patients with non-alcoholic fatty liver disease (NAFLD)^20^. However, limited reports, if any, have directly investigated the modifications induced by DAAs on SCFA-producing bacteria and SCFA production in patients with chronic HCV infection.

At baseline, our data showed that certain SCFAs-producing taxa were significantly low in patients with chronic HCV infection, indicating the depletion of SCFA-producing bacteria was present in untreated individuals. Following successful DAA therapy, we demonstrated that *Blautia, Bifidobacterium, Subdoligranulum and Fusicatenibacter* and others were enriched compared to those of before treatment. Among them, *Bifidobacterium* is one of the most well-established probiotics that provides positive health benefits through several mechanisms, including modulation of host immunity, improvement of intestinal integrity and reduction in intestinal endotoxins^21^. *Bifidobacterium* is a main butyrate producer that also plays protective roles in liver injury and decreased abundance of this bacterium could enhance liver inflammation and contribute to the progressive disease in NAFLD^22^. A recent report also demonstrated that *Bifidobacterium* was significantly decreased in HBV-related cirrhosis compared with healthy controls^23^. In HCV, the progression of fibrosis and cirrhosis in infected individuals also appeared to be related to altered *Bifidobacterium* composition^17, 24^. Regarding *Subdoligranulum*, a strictly anaerobic, butyrate-producing bacterium, was negatively linked to different parameters associated with metabolic risks in patients with NAFLD^25^. Additionally, *Subdoligranulum* was found to be less abundance in patients with alcoholic liver disease, as well as in cirrhotic patients compared to healthy individuals^26^.

To gain further insight into the mechanisms by which SCFAs might involve in the pathogenesis of chronic HCV infection, we subsequently analyzed the alteration of circulating LBP, a surrogate marker of microbial translocation. LBP is a 50-kD polypeptide synthesized by the hepatocytes, which binds the lipid A portion of lipopolysaccharide (LPS) and induces signal pathways related to inflammatory responses^27^. Comparing to LPS, LBP has gained interest as an alternative biomarker of microbial translocation due to its longer half-life and more stable in circulation. In this study, our data showed that baseline LBP levels were increased in patients with chronic HCV infection compared with healthy controls. Notably, elevated LBP was observed not only in advanced liver fibrosis, but also found in early fibrosis stages, suggesting that microbial translocation exists throughout the course of chronic HCV infection^10, 11^. In addition, we found that plasma LBP levels significantly declined in HCV-infected patients after successful DAA therapy. This was in line with previous reports demonstrating that there was a significant reduction in plasma biomarkers of microbial translocation after DAA treatment even though their values were not normalized^12, 13^. Interestingly, although baseline LBP levels did not correlate with fecal BCoAT, their changes between post- and pre-treatment (Δ) were found to have significant correlation in our study. Together, it could indicate from our data that HCV therapy alters the composition of gut microbiota by enriching SCFA-producing bacteria, which in turn leads to increased SCFA production and improved microbial translocation.

Elevated plasma level of intestinal fatty acid binding protein (I-FABP) is considered as a surrogate marker of intestinal damage and is associated with disease outcome in patients with chronic HCV infection^10^. In this report, our data showed that I-FABP level at baseline was significantly higher in HCV-infected patients when compared to healthy individuals. Interestingly, increased plasma levels of I-FABP were comparable between patients with HCV monoinfection and HCV/HIV coinfection. These data suggested that gut barrier defect could be identified in most HCV-infected patients, irrespective of HIV status. Following successful DAAs, however, plasma I-FABP concentrations were not declined significantly in responders. These results were opposed to previous data regarding interferon-based therapy in patients with chronic HCV infection^10^, but were similar to recent evidence indicating that successful DAA therapy was not associated with the modifications in the intestinal barrier function^12^. Thus, it is likely that microbial translocation might be, at least in part, linked to altered specific composition of gut microbiota, rather than related to gut barrier dysfunction after DAA therapy. Alternatively, these observations may be associated with the improvement of liver function following achieved SVR, which results in enhanced production of bile acids that could exert direct antimicrobial defense and ameliorate bacterial translocation via farnesoid X receptor (FXR) signaling^28^.

Of note, it was observed that significantly improvement of gut dysbiosis, as well as the enhancement of fecal BCoAT and decreased LBP were predominantly found in HCV-monoinfected patients compared with those of HCV/HIV coinfected individuals. Current data indicate that HIV infection has been associated with alterations in gut microbiota composition and related microbial metabolites that are not fully restored with effective antiretroviral therapy (ART)^29^. Despite suppressive ART, most studies show that HIV-infected individuals typically have persistent gut dysbiosis associated with decreased bacterial richness and diversity. In fact, altered microbiota-related products in HIV-infected individuals such as SCFAs and LPS have been associated with the development of leaky gut syndrome, which promotes microbial translocation leading to persistent immune dysfunction and chronic inflammation^30^. Thus, gut dysbiosis in ART-treated individuals has been continuing linked to disease progression of HIV and its co-morbidities including metabolic disorders and cardiovascular disease.

Regarding HCV/HIV coinfection, this disease entity is considered as a more complex that gut microbiota and related microbial metabolites altered by both HCV and HIV, and therefore, warrants further concern. With the advent of highly effective DAAs, HCV treatment has been changed as HCV mono- and HCV/HIV coinfected individuals could achieve similar SVR rates of over 95%^3^. Moreover, data from several cohorts have indicated a clear benefit of HCV cure in terms of liver fibrosis regression and reducing the risk of developing HCC, regardless of HIV status. Nevertheless, long-term improvement of gut dysbiosis and related metabolites after DAA treatment in patients with HCV/HIV coinfection has rarely been investigated. Thus, in this study, we further explored the impact of DAAs on microbiota restoration and related metabolites in patients with HCV/HIV coinfection compared with the mono-infected group. Our findings demonstrated that HCV clearance could, to some extent, restore gut dysbiosis and SCFA production, particularly in coinfected individuals with mild liver fibrosis. These findings suggest that despite improvement in liver fibrosis, gut dysbiosis and microbial translocation might persist after SVR in coinfected patients. Considering the fact that DAAs could not fully restore gut microbiota in coinfected individuals, gut dysbiosis is likely to remain a prominent risk in the context of HCV/HIV coinfection after HCV cure. Thus, additional therapeutic interventions to attenuate HIV-associated gut dysbiosis might be required. For instance, a recent pilot placebo-controlled study has demonstrated that repeated oral capsular fecal microbiota transplantation (FMT) is a feasible strategy to restore gut health in individuals with HIV infection^31^.

Although this study was mainly based on associations/correlations that might not prove a cause-and-effect relationship, our data had several strengths to be mentioned. This is the first report that longitudinally investigates the effect of DAAs on gut microbiota in comparison of patients with HCV mono- and HCV/HIV coinfection. Unlike previous reports, our study recruited a large number of patients across different stages of liver fibrosis, who were treated with the same DAAs and thus excluded confounding effects associated with different antiviral regimens. Additionally, we directly compared the alteration of microbial community structure in both responders and non-responders as it would verify that alleviated gut dysbiosis was related to treatment response. Despite these advantages, the study had some limitations, a relatively small number of co-infected patients, particularly those with significant/advanced fibrosis. Thus, it would be necessary to further assess the impact of DAAs on gut microbiota restoration in a larger sample size of HCV/HIV coinfected patients.

**Figure 7:**
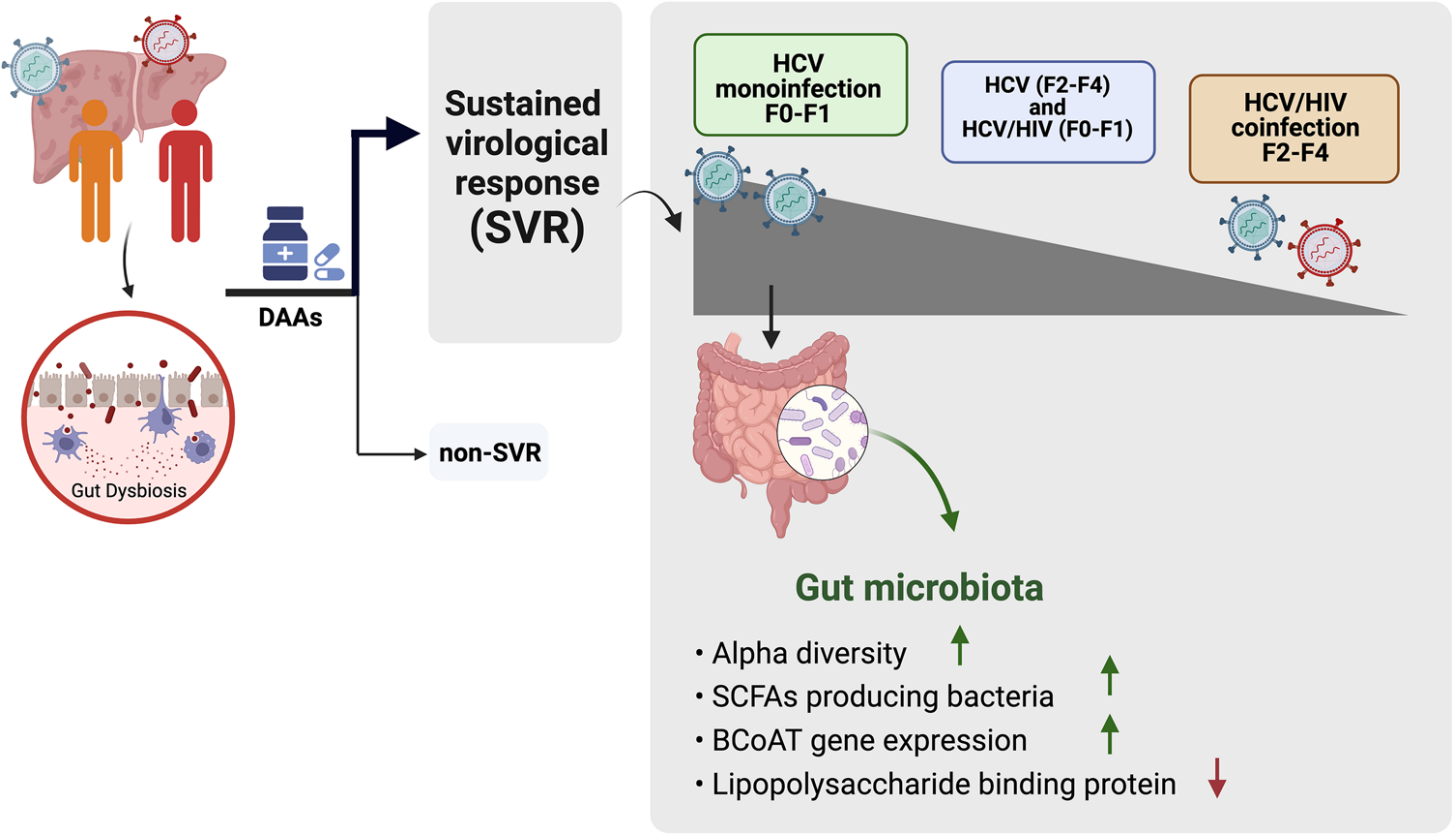
Overall conclusion of the effect of long-term DAAs treatment on gut microbiota, BCoAT gene and microbial translocation in patients with HCV monoinfection and HCV/HIV coinfection.

In conclusion, our long-term results indicated that HCV cure could result in the restoration of gut microbiota composition and SCFA production, which linked to the improvement of microbial translocation marker, particularly in HCV-monoinfected patients with low-grade fibrosis (Fig. 7). According to clinical practice guidelines, HCV treatment with DAAs should be recommended for all eligible patients without delay, irrespective of fibrosis stages. In this context, our study provides additional evidence supporting early HCV treatment to optimize the restoration of gut microbiota, which in turn will lead to the prevention of long-term hepatic and extra-hepatic complications related to gut dysbiosis.

## Materials and methods

### Patients

The patient group has been described before^14^. In brief, patients with HCV monoinfection and HCV/HIV coinfection treated with an elbasvir-grazoprevir (EBR/GZR) combination, the study subjects were recruited for clinical trials (clinicaltrials.gov; NCT03037151) between August 2018 and April 2019 at the King Chulalongkorn Memorial Hospital, Bangkok, Thailand. Initially, sixty-two patients with HCV monoinfection and twenty-four patients with HCV/HIV coinfection were enrolled in this study. Inclusion criteria for the chronic HCV infection group were patients who were newly diagnosed with HCV genotype 1 with no previous record of HCV treatment and were potentially eligible to enroll in this study.

For HCV/HIV coinfection, has previously received antiretroviral therapy (ART), and undetectable plasma HIV-RNA levels will be included. This study excluded participants co-infected with hepatitis B virus (HBV), had other liver diseases, including fatty liver disease and alcohol liver disease, received prior treatment, and were diagnosed with decompensated cirrhosis and HCC. As controls, twenty healthy individuals without any liver and metabolic diseases were included in this study.

After follow-up at week 72, fifty patients with HCV monoinfection and nineteen patients with HCV/HIV coinfection remained in this study. All participants obtained written informed consent under the Institutional Review Board of the Faculty of Medicine, Chulalongkorn University (IRB No.378/61). Furthermore, the protocol was conducted according to the Helsinki Declaration and Good Clinical Practice Guidelines of the International Conference of Harmonization (ICH GCP) and approved by the Research Ethics Review Committee for Research Involving Human Subjects, Chulalongkorn University.

### Clinical data collection

Demographic data of all participants were collected, including gender, age, and body mass index (BMI). All participants who enrolled in this study measured liver stiffness (LS) by magnetic resonance elastography (MRE) using the MR imaging system Philips Ingenia at 3.0 T (Philips Healthcare, Best, the Netherlands) to determine fibrosis stage^32^. An average value from the three measurement slices was used to determine LS. The cut-off values of LS are 3.2 kPa for significant fibrosis (≥ F2), 4.0 kPa for advanced fibrosis (≥ F3), and 4.6 kPa for cirrhosis (F4)^33^. In this study, patients were allocated into 2 groups: no or early fibrosis (F0–F1) and significant fibrosis to cirrhosis (F2–F4).

### Fecal collection and microbial DNA extraction

Patients were asked to stop antibiotics, prebiotics and probiotics supplement, or proton pump inhibitors (PPIs) within 2 weeks before acceptance and during the study period. Participants collected fecal samples at into tubes with DNA stabilizer (DNA/RNA Shield™ Fecal Collection Tube) and kept at − 80 °C until further microbiota analyses. The procedure was done following the standard protocol from the International Human Microbiome Standard (IHMS)^8^. Total microbial DNA from feces were extracted using Quick-DNA™ Fecal/Soil Microbe Miniprep Kit (Zymo Research Corp.) according to the manufacturer’s protocol. DNA concentration and purity was measured by DeNovix™ UV–Vis spectrophotometer and will be stored at − 20 °C until perform the sequencing.

### 16S rRNA sequencing and Bioinformatics Analysis

The V3–V4 hypervariable regions of the 16S rRNA gene were targeted using the forward primer (341F) and reverse primer (805R)^23^. DNA libraries were constructed, and paired-end sequencing was performed on an Illumina MiSeq 300 bp platform (Illumina, San Diego, CA, USA) by Génome Québec Innovation Centre (Montréal, QC, Canada). The raw reads were demultiplexed and removed non-biological nucleotide using Cutadapt version 2.8 to create an amplicon sequence variant (ASV) table by using the DADA2 pipeline^34, 35^. Silva version 138.1 was achieved to assign taxonomy^36^. R studio with in-house script calculated relative abundance and richness, alpha-diversity, including Chao1and Simpson indices, using the Phyloseq R package (v.1.38.0)^37^. For data visualization, beta-diversity was estimated by calculating Bray–Curtis distances and then visualized means of Principal Coordinate analysis (PCoA) by MicrobiomeAnalyst web-based platform (https://www.microbiomeanalyst.ca/). The composition bar plot was generated with the ggplot2 R package (v.3.3.5). The raw sequencing data have been deposited in NCBI SRA (https://www.ncbi.nlm.nih.gov/sra) under the accession number PRJNA882461. The information number of raw reads and processed read is summarized in Table S1.

### Quantification of butyryl-CoA:acetateCoA transferase (BCoAT) gene

Quantification of gut microbiome metabolite gene (BCoAT) and V3–V4 16S gene (total bacteria) was performed by qPCR using 4X CAPITAL™ qPCR Green Master Mix (Biotech Rabbit). The degenerate primers were amplified BCoAT gene^38^ using the forward primer: 5′-GCIGAICATTTCACITGGAAYWSITGGCAYATG-3′ and reverse primer: 5′-CCTGCCTTTGCAATRTCIACRAANGC-3′, and V3–V4 16S rRNA gene^39^. The qPCR conditions started with a DNA-denaturation step at 95 °C for 15 min, followed by 40 cycles of denaturation at 95 °C for 15 s, annealing at a primer-specific temperature for 20 s, extension at 72 °C for 30 s followed by the detection with a specific temperature. Standard curves ranging from 10^2^ to10^7^ copies (10-log-fold) were produced using the V3–V4 of 16S rRNA gene and BCoAT amplicons from the fecal DNA sample. The standard curve was measured by absolute quantifications of the BCoAT and V3–V4 genes copy numbers. BCoAT copy numbers were normalized with V3–V4 copy numbers in each sample.

### Microbial surrogate biomarkers analysis

Peripheral blood samples were collected and processed within 2 h for plasma separation and stored at − 80 °C until further analysis. Lipopolysaccharide Binding Protein (LBP) and Intestinal fatty acid binding protein (I-FABP) were determined by an enzyme-linked immunosorbent assay kit (Hycult Biotech, Uden, The Netherlands) according to the manufacturer's instructions. The dilution of plasma samples was 1:1000 for LBP and 1:2 for I-FABP, as recommended in the instructions.

### Statistical analysis

Statistical analysis of clinical parameters was achieved using IBM SPSS version 22.0.0 (IBM SPSS, Inc, Chicago, IL, USA). The normality test was performed by using the Kolmogorov–Smirnov test. To compare more than two groups and categories data, one-way ANOVA and Chi-square test were used. Independent data between 2 groups was performed using Student’s t-test and Mann–Whitney test. The correlation between the BCoAT gene, microbes, and clinical parameters was calculated using Spearman Rank Correlation. The *P*-value < 0.05 was considered statistically significant. The PCoA plot based on Bray–Curtis was determined the significance by using PERMANOVA analyzed by Microbiome Analyst web-based platform (https://www.microbiomeanalyst.ca/).

### Supplementary Information

Supplementary Information.

## Data Availability

The datasets generated during the current study are available in the NCBI Sequence Read Archive (SRA) database, BioProject: PRJNA882461 (https://www.ncbi.nlm.nih.gov/bioproject/PRJNA882461).
