# Supplementary material for: Long-term benefit of DAAs on gut dysbiosis and microbial translocation in HCV-infected patients with and without HIV coinfection
Source: Sci Rep. 2023 Sep 2;13:14413. doi: 10.1038/s41598-023-41664-7 (PMC10475021; doi:10.1038/s41598-023-41664-7)
Supplement: Supplementary file 1 — Supplementary Information. [file 41598_2023_41664_MOESM1_ESM.pdf]

Supporting information

Long-term Benefit of DAAs on Gut Dysbiosis and Microbial Translocation in HCV-infected Patients with and without HIV Coinfection

Natthaya Chuaypen[1#], Thananya Jinato[1,2#], Anchalee Avihingsanon[3], Intawat Nookaew[4], Yasuhito Tanaka[5], Pisit Tangkijvanich[1]*

[1]Center of Excellence in Hepatitis and Liver Cancer, Department of Biochemistry, Faculty of Medicine, Chulalongkorn University, Bangkok, Thailand

[2]Doctor of Philosophy Program in Medical Sciences, Graduate Affairs, Faculty of Medicine, Chulalongkorn University, Bangkok, Thailand

[3]The HIV Netherlands Australia Thailand Research Collaboration (HIV-NAT), Bangkok, Thailand

[4]Department of Biomedical Informatics, College of Medicine, University of Arkansas for Medical Sciences, Little Rock, AR, USA

[5]Division of Integrated Medical and Pharmaceutical Sciences, Department of Gastroenterology and Hepatology, Faculty of Life Sciences, Kumamoto University, Kumamoto, Japan

#Co-first author

*Corresponding author

Prof. Pisit Tangkijvanich, M.D.

Center of Excellence in Hepatitis and Liver Cancer, Faculty of Medicine, Chulalongkorn University, Bangkok, Thailand

Email: pisittkvn@yahoo.com

Supplementary Figure S1. Alpha diversity of gut microbiome (Simpson) at baseline and follow-up week-72 (FUw72) in (a) healthy controls, patients with SVR and non-SVR, (b) F0-F1 vs. F2-F4 fibrosis stages, (c) HCV monoinfection vs. HCV/HIV coinfection, (d) subgroup analysis of fibrosis stages and HIV status.

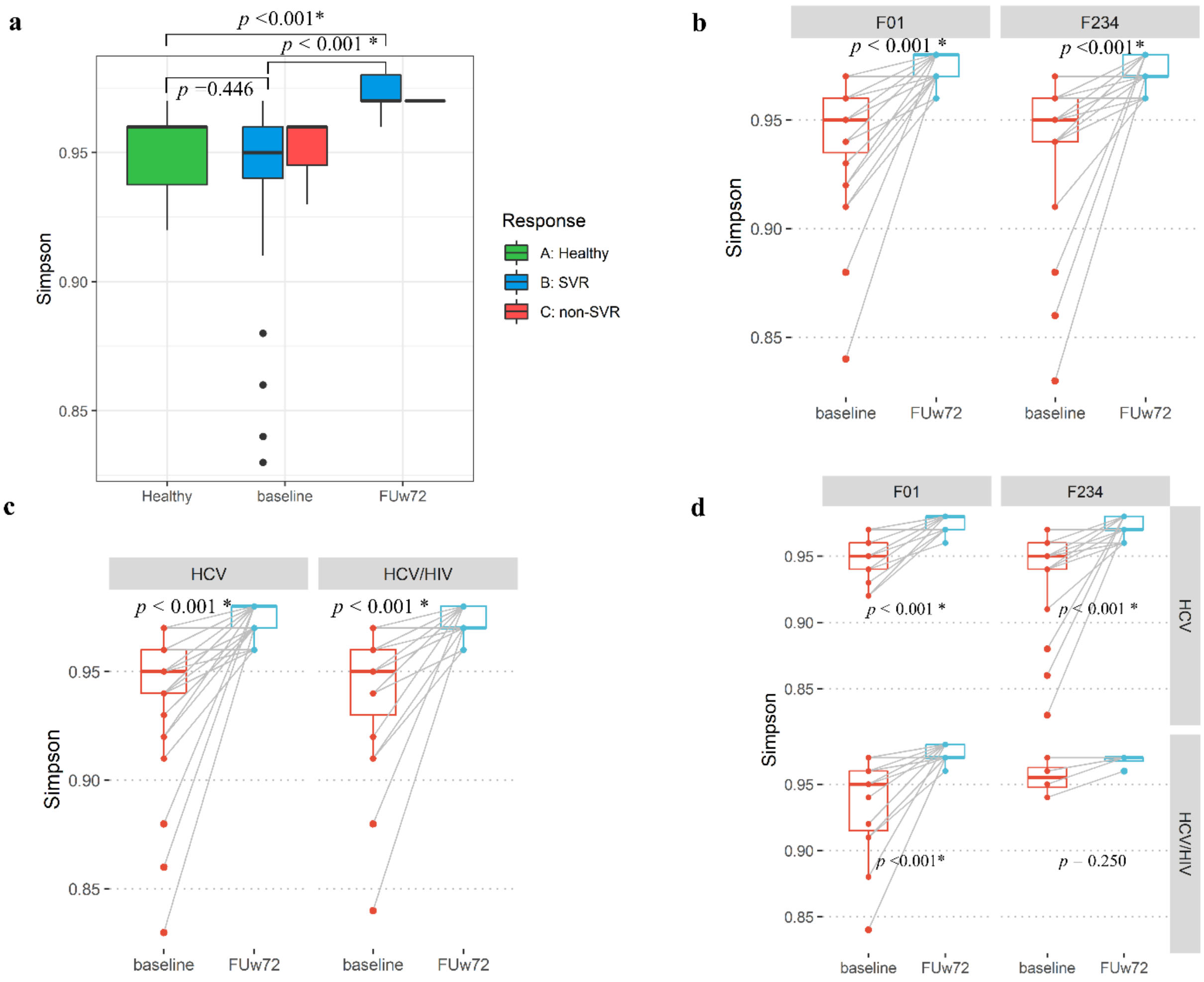

Supplementary Figure S2. The correlation between the change of BCoAT gene level and plasma LBP level in patients at baseline and follow-up week-72 (FUw72) in (a) overall patients, (b) HCV monoinfection, (c) HCV/HIV coinfection.

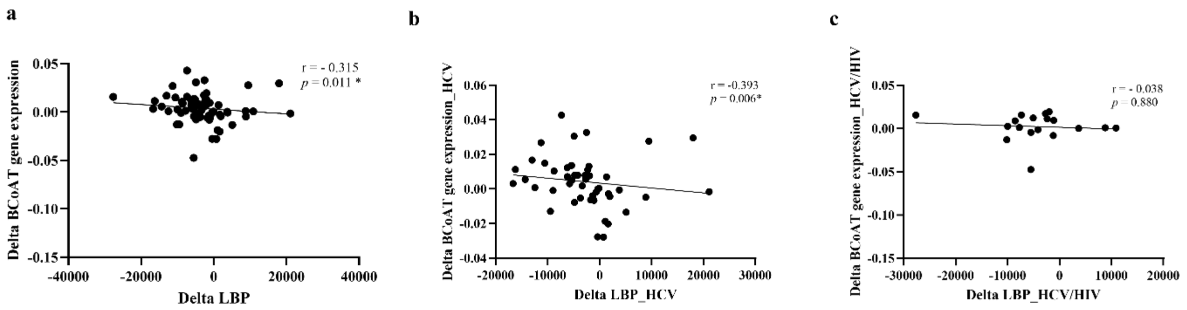

Table S1. The summary table of number of read processing

SampleID	Group	input	filtered	denoisedF	denoisedR	merged	nonchim
H12	Healthy	45420	39364	38814	39197	38384	38030
H13	Healthy	44464	38142	37778	37944	37252	37094
H14	Healthy	55494	48594	48191	48397	47756	47472
H15	Healthy	26276	21270	21122	21213	20929	20914
H16	Healthy	26423	22950	22750	22797	22458	22264
H20	Healthy	35686	30801	30520	30597	30082	29452
H21	Healthy	16896	14875	14681	14768	14543	14387
H23	Healthy	64442	53035	52465	52794	51948	45726
H26	Healthy	23922	20212	19936	19984	19542	19493
H27	Healthy	48183	38648	37661	38330	36885	35351
H28	Healthy	51445	44765	44223	44544	43499	43138
H29	Healthy	68397	59110	58109	58614	56554	52729
H30	Healthy	43975	37572	36562	37126	35370	32529
H32	Healthy	30200	26247	26045	26141	25793	24882
H2	Healthy	27419	23909	23626	23739	23287	23263
H5	Healthy	43520	38008	37435	37788	36492	34620
H7	Healthy	44557	36014	35588	35793	35072	34699
H9	Healthy	14587	11120	10921	11021	10605	10550
H10	Healthy	28368	22521	22327	22389	22028	21976
H11	Healthy	49569	40177	39398	39788	38230	35361
A10	HCV	41971	29894	29806	29782	29331	28482
A11	HCV	54247	38558	38064	38093	35050	33323
A14	HCV	49900	34863	34302	34053	32401	32337
A15	HCV	58328	41283	40811	40916	38186	34752
A16	HCV	41614	29775	29628	29484	28981	28964
A17	HCV	59146	41235	40948	40919	39917	39825
A18	HCV	54175	37952	37719	37527	36564	36326
A19	HCV	49458	34687	34377	34297	33181	33087
A20	HCV	58565	41474	41350	41336	40994	38675
A21	HCV	55220	38576	38336	38302	37478	37373
A22	HCV	63731	45202	44710	44076	40491	37835
A23	HCV	51819	35433	35169	35246	34522	34223
A24	HCV	64188	45188	44835	44856	43084	42847
A25	HCV	56218	39629	39441	39447	38607	38195
A26	HCV	48538	34293	33995	33973	33307	33288
A27	HCV	64835	46444	46053	45975	42400	40217
A28	HCV	67732	48222	47738	47126	43717	40674
A29	HCV	55307	39188	38794	38902	37622	37198
A2	HCV	53596	36441	35860	34413	29267	27184
A30	HCV	48292	34241	33962	33903	33002	32906

A31	HCV	41962	29740	29492	29405	28763	28741
A32	HCV	50183	35894	35705	35549	35030	32011
A34	HCV	55541	38429	38228	37858	37109	37091
A36	HCV	67810	47213	46458	46214	42314	41913
A37	HCV	57004	40355	40106	39741	39205	39108
A38	HCV	53335	37953	37789	37622	36694	36633
A39	HCV	55305	39210	38960	38585	36719	35706
A3	HCV	37423	26467	26222	25826	24891	23908
A40	HCV	56147	39564	39330	38284	37775	37172
A41	HCV	43234	30712	30519	30382	29677	29628
A42	HCV	58530	41536	40917	40525	36544	34241
A43	HCV	52101	36963	36522	36262	32754	29739
A44	HCV	54874	38014	37717	37501	36522	35905
A45	HCV	59165	42178	41836	41512	39461	37924
A46	HCV	50204	35588	35218	35155	34274	33010
A47	HCV	54608	38838	38461	38638	36804	32315
A48	HCV	58721	41860	41388	41300	37918	34789
A49	HCV	48610	34082	33810	33759	33166	32640
A4	HCV	44253	31682	31518	31050	30059	29874
A51	HCV	52310	36291	35862	35883	34734	34574
A53	HCV	52529	36859	36685	36676	36003	34761
A54	HCV	63635	44732	43949	43940	39379	37979
A55	HCV	58466	46166	45963	45753	44592	44500
A57	HCV	51132	35681	35383	35198	34263	33127
A5	HCV	39675	28485	28283	27587	26918	26895
A61	HCV	48997	34628	34269	33560	32658	32562
A63	HCV	57895	41211	40995	40793	39737	39406
B1	HCV/HIV	54121	38390	38193	38218	37485	30724
B2	HCV/HIV	52425	37414	37229	37163	36429	35943
B3	HCV/HIV	54941	38157	37794	37541	36069	36058
B4	HCV/HIV	55216	39512	39128	38953	36547	35792
B5	HCV/HIV	56978	40546	40401	40276	39570	38765
B6	HCV/HIV	60984	43417	43295	43180	42688	42448
A6	HCV	46935	33888	33691	33075	32118	32107
B7	HCV/HIV	55122	38489	38291	38274	37236	37176
B8	HCV/HIV	54888	38864	38603	38509	37077	36106
B9	HCV/HIV	64524	45841	45407	45443	43908	43543
B12	HCV/HIV	55247	38484	38169	37610	35429	32956
B14	HCV/HIV	46611	32984	32822	32756	32043	30085
B16	HCV/HIV	48440	33879	33524	33317	31977	31885
B17	HCV/HIV	48856	35000	34587	34177	31765	30341
B18	HCV/HIV	45746	32153	31813	31805	30897	30658
A7	HCV	51694	37047	36496	35740	31927	31238

B22	HCV/HIV	50742	35830	35629	35008	34441	34196
B25	HCV/HIV	54160	38170	37978	37941	37370	37000
B26	HCV/HIV	66846	47644	47495	47207	46408	46398
B27	HCV/HIV	57017	40057	39693	39768	38928	37615
B31	HCV/HIV	74746	52504	51585	51516	46826	46172
A9	HCV	59906	43102	42654	42656	40335	36868
D1	HCV_SVR72	77331	67518	60379	65235	44205	31429
D13	HCV_SVR72	49322	43259	38953	42205	32651	26342
D25	HCV_SVR72	49657	42910	35964	41700	29013	20029
D37	HCV_SVR72	55060	48490	41390	46955	31669	23147
D49	HCV_SVR72	47624	42295	37456	41321	32458	28349
G11	HCV/HIV_SVR72	75268	65867	55665	64516	48181	44937
D2	HCV_SVR72	48362	41972	37452	40149	27011	23033
D14	HCV_SVR72	49956	42998	37841	41658	30555	26611
D26	HCV_SVR72	47772	42038	38626	41277	33927	29331
D38	HCV_SVR72	51970	45881	41236	43783	30069	25357
D51	HCV_SVR72	49460	43976	34375	43209	29604	22842
G12	HCV/HIV_SVR72	53870	46216	38891	42792	29803	21791
D3	HCV_SVR72	31170	27820	26495	27620	25825	25759
D15	HCV_SVR72	52623	45890	40760	43783	29840	25876
D27	HCV_SVR72	48241	42621	36243	41542	28502	20217
D39	HCV_SVR72	52466	45711	39889	44665	31453	23546
G1	HCV/HIV_SVR72	52696	45693	42049	43722	32094	25722
G13	HCV/HIV_SVR72	52266	45900	42559	44329	31963	25351
D4	HCV_SVR72	70174	61475	52253	60487	45995	36085
D16	HCV_SVR72	46037	39522	35758	38540	30237	23139
D28	HCV_SVR72	49885	44024	40449	42781	30335	23882
D40	HCV_SVR72	48345	42124	36729	40660	29048	24779
G2	HCV/HIV_SVR72	53931	46447	38914	44840	29810	21549
G14	HCV/HIV_SVR72	77631	67640	56036	64232	43762	31207
D5	HCV_SVR72	37473	33016	29049	32113	24654	22032
D17	HCV_SVR72	51930	45300	41554	43028	30704	24276
D29	HCV_SVR72	49412	43293	40657	42236	33699	27742
D41	HCV_SVR72	44309	38303	35053	36575	26064	21274
G3	HCV/HIV_SVR72	46557	41198	37895	40419	33375	31058
G15	HCV/HIV_SVR72	53437	47009	41439	45299	32144	23942
D6	HCV_SVR72	49937	43915	38885	42612	31151	23438
D18	HCV_SVR72	49907	44179	40652	42557	31905	24085
D30	HCV_SVR72	53512	46524	42610	44986	33157	24988
D42	HCV_SVR72	48974	42968	39592	41832	34153	28849
G4	HCV/HIV_SVR72	42963	37298	33215	36022	25343	18958
G16	HCV/HIV_SVR72	48068	42066	37319	40687	29046	20761
D7	HCV_SVR72	49043	42098	37266	40367	27786	20500

D19	HCV_SVR72	52222	46059	41837	44196	30938	24457
D31	HCV_SVR72	48111	41805	36038	39660	26927	23903
D43	HCV_SVR72	55791	49567	42917	46427	32914	23539
G5	HCV/HIV_SVR72	49284	43153	36971	41886	28266	21119
G18	HCV/HIV_SVR72	52518	45944	38780	44889	31537	19849
D8	HCV_SVR72	55269	48215	44001	45941	31294	25191
D20	HCV_SVR72	46685	41225	39520	40591	36538	35999
D32	HCV_SVR72	49901	44086	37348	42903	30156	22712
D44	HCV_SVR72	42962	37826	31072	35818	26896	25148
G6	HCV/HIV_SVR72	40511	35464	30164	35142	28998	28527
G19	HCV/HIV_SVR72	51312	44915	38590	43477	28982	20523
D9	HCV_SVR72	47043	41020	32922	40232	29300	28418
D21	HCV_SVR72	44623	39399	36561	38103	29444	26365
D33	HCV_SVR72	51292	44522	41110	42803	29327	21530
D45	HCV_SVR72	50162	43619	38817	42494	29650	20561
G7	HCV/HIV_SVR72	46748	40587	37077	38736	27329	22565
G20	HCV/HIV_SVR72	52045	44982	41315	43595	32720	26024
D10	HCV_SVR72	47921	42694	37636	41848	32201	27653
D22	HCV_SVR72	52705	46315	43379	45185	36239	30763
D34	HCV_SVR72	46142	39775	34831	37755	26072	22747
D46	HCV_SVR72	50108	44171	41426	42727	31543	24357
G8	HCV/HIV_SVR72	49068	42938	38566	39687	28869	21446
D11	HCV_SVR72	66082	58736	51191	58131	47670	46708
D23	HCV_SVR72	46864	40766	36616	39910	32406	30580
D35	HCV_SVR72	49478	43686	40475	42519	34583	30912
D47	HCV_SVR72	49789	43860	40858	42777	34553	26620
G9	HCV/HIV_SVR72	51060	45055	40922	43051	30119	24740
D12	HCV_SVR72	48882	42975	39426	42034	35049	33172
D24	HCV_SVR72	53400	46569	41378	44639	30046	21437
D36	HCV_SVR72	73292	62858	55469	59770	38266	29925
D48	HCV_SVR72	27374	23934	22436	23711	21778	21730
G10	HCV/HIV_SVR72	48971	41932	37159	40713	29367	21864

Table S2. Gut microbiota composition at baseline

Baseline	Healthy(n=20)		HCV(n=50)		HCV/HIV(n=19)		Asymp. Sig. (2-tailed)
	**median**	**IQR**	**median**	**IQR**	**median**	**IQR**	
Blautia	0.022	0.015-0.037	0.021	0.013-0.040	0.013	0.009-0.019	0.017b,0.015c
Bifidobacterium	0.001	0.000-0.010	0.003	0.001-0.011	0.001	0.000-0.002	0.027c
Collinsella	0.004	0.002-0.012	0.000	0.000-0.001	0.000	0.000-0.001	<0.001a, b
UCG-002	0.014	0.005-0.035	0.003	0.000-0.017	0.004	0.001-0.033	0.013a
Alistipes	0.015	0.004-0.025	0.014	0.002-0.029	0.004	0.001-0.014	0.030b
Subdoligranulum	0.010	0.003-0.022	0.004	0.000-0.013	0.002	0.000-0.004	0.029a, <0.001b
[Ruminococcus] torques group	0.004	0.002-0.011	0.006	0.002-0.011	0.002	0.001-0.006	0.015c
Fusicatenibacter	0.005	0.003-0.010	0.004	0.001-0.010	0.001	0.000-0.002	<0.001b,0.004c
Lachnospira	0.019	0.006-0.030	0.009	0.003-0.020	0.006	0.001-0.011	0.035a,0.003b
Alloprevotella	0.000	0.000-0.000	0.000	0.000-0.000	0.016	0.000-0.039	0.029b, <0.001c
Succinivibrio	0.000	0.000-0.000	0.000	0.000-0.000	0.000	0.000-0.023	0.007c
Parabacteroides	0.009	0.007-0.014	0.006	0.002-0.010	0.005	0.003-0.016	0.031a
Coprococcus	0.006	0.003-0.010	0.004	0.000-0.008	0.002	0.001-0.005	0.016b
Lachnospiraceae NK4A136 group	0.011	0.005-0.029	0.003	0.001-0.013	0.010	0.003-0.013	0.009a
Romboutsia	0.001	0.000-0.001	0.000	0.000-0.001	0.001	0.000-0.001	0.025a
Catenibacterium	0.000	0.000-0.000	0.000	0.000-0.002	0.000	0.000-0.002	0.040b
CAG-352	0.000	0.000-0.020	0.000	0.000-0.000	0.000	0.000-0.000	0.019a
Christensenellaceae R7 group	0.003	0.000-0.007	0.000	0.000-0.002	0.000	0.000-0.014	0.003a
Lachnospiraceae UCG-004	0.005	0.002-0.012	0.002	0.000-0.005	0.002	0.001-0.003	0.038a,0.024b
Clostridium sensu tricto-1	0.002	0.000-0.005	0.000	0.000-0.002	0.000	0.000-0.001	0.013a,0.007b
Erysipelotrichaceae UCG-003	0.003	0.001-0.004	0.000	0.000-0.001	0.000	0.000-0.001	0.002a, <0.001b
Lachnospiraceae ND3007 group	0.002	0.000-0.003	0.001	0.000-0.001	0.000	0.000-0.001	0.037a,0.003b
a=Healthy vs. HCV monoinfection, b=Healthy control vs. HCV/HIV coinfection, c=HCV vs. HCV/HIV coinfection							

Table S3. Relative abundance (>1%) at genus levels in baseline and follow-up week-72 (FUw72) of HCV monoinfection

HCV monoinfection	Baseline	FUw72	*P*-values
Bacteroides	0.180±0.32	0.110±0.13	< 0.001
Blautia	0.023±0.03	0.077±0.04	< 0.001
Bifidobacterium	0.002±0.01	0.030±0.05	0.001
Lachnoclostridium	0.013±0.03	0.009±0.02	0.040
Collinsella	0.000±0.00	0.041±0.02	< 0.001
Subdoligranulum	0.004±0.01	0.014±0.02	0.001
Ruminococcus torques group	0.005±0.01	0.014±0.02	< 0.001
Fusicatenibacter	0.003±0.01	0.014±0.02	< 0.001
Dorea	0.005±0.00	0.014±0.01	< 0.001
Eubacterium hallii group	0.004±0.01	0.013±0.01	< 0.001

Data shown in median ± IQR, P-values (Wilcoxon Signed Ranks Test)

Table S4. Relative abundance (>1%) at genus levels in baseline and follow-up week-72 (FUw72) of HCV/HIV coinfection

HCV/HIV co-infection	Baseline	FUw72	*P*-values
Bacteroides	0.134±0.44	0.059±0.15	0.036
Blautia	0.013±0.01	0.065±0.02	< 0.001
Prevotella	0.041±0.14	0.000±0.05	0.049
Agathobacter	0.012±0.02	0.023±0.03	0.040
Bifidobacterium	0.001±0.00	0.026±0.08	0.001
Megamonas	0.000±0.01	0.040±0.1	0.004
Collinsella	0.000±0.00	0.043±0.02	< 0.001
Ruminococcus torques group	0.002±0.01	0.011±0.02	0.006
Dorea	0.004±0.00	0.016±0.01	< 0.001
Eubacterium hallii group	0.003±0.00	0.014±0.01	< 0.001
Coprococcus	0.002±0.00	0.01±0.02	0.028
Lachnospiraceae NK4A136 group	0.010±0.01	0.000±0.00	0.001

Data shown in median ± IQR, P-values (Wilcoxon Signed Ranks Test)
